# Effect of Micro- and Nano-Lignin on the Thermal, Mechanical, and Antioxidant Properties of Biobased PLA–Lignin Composite Films

**DOI:** 10.3390/polym14235274

**Published:** 2022-12-02

**Authors:** Sofia P. Makri, Eleftheria Xanthopoulou, Panagiotis A. Klonos, Alexios Grigoropoulos, Apostolos Kyritsis, Konstantinos Tsachouridis, Antonios Anastasiou, Ioanna Deligkiozi, Nikolaos Nikolaidis, Dimitrios N. Bikiaris

**Affiliations:** 1 Creative Nano PC, 43 Tatoiou, Metamorfosi, 14451 Athens, Greece; 2 Laboratory of Polymer Chemistry and Technology, Department of Chemistry, Aristotle University of Thessaloniki, 54124 Thessaloniki, Greece; 3 Department of Physics, National Technical University of Athens (NTUA), Zografou Campus, 15780 Athens, Greece; 4 Department of Chemical Engineering and Analytical Science, University of Manchester, Manchester M1 3AL, UK

**Keywords:** poly(lactic acid), PLA, lignin, nanolignin, composite films, nucleation, mechanical properties, antioxidant activity, food packaging

## Abstract

Bio-based poly(lactic acid) (PLA) composite films were produced using unmodified soda micro- or nano-lignin as a green filler at four different contents, between 0.5 wt% and 5 wt%. The PLA–lignin composite polymers were synthesized by solvent casting to prepare a masterbatch, followed by melt mixing. The composites were then converted into films, to evaluate the effect of lignin content and size on their physicochemical and mechanical properties. Differential scanning calorimetry (DSC), supported by polarized light microscopy (PLM), infrared spectroscopy (FTIR-ATR), X-ray diffraction (XRD), and transmission electron microscopy (TEM) were employed to investigate the PLA crystallization and the interactions with Lignin (L) and Nanolignin (NL). The presence of both fillers (L and NL) had a negligible effect on the glass transition temperature (chain diffusion). However, it resulted in suppression of the corresponding change in heat capacity. This was indicative of a partial immobilization of the PLA chains on the lignin entities, due to interfacial interactions, which was slightly stronger in the case of NL. Lignin was also found to facilitate crystallization, in terms of nucleation; whereas, this was not clear in the crystalline fraction. The addition of L and NL led to systematically larger crystallites compared with neat PLA, which, combined with the higher melting temperature, provided indications of a denser crystal structure in the composites. The mechanical, optical, antioxidant, and surface properties of the composite films were also investigated. The tensile strength and Young’s modulus were improved by the addition of L and especially NL. The UV-blocking and antioxidant properties of the composite films were also enhanced, especially at higher filler contents. Importantly, the PLA–NL composite films constantly outperformed their PLA–L counterparts, due to the finer dispersion of NL in the PLA matrix, as verified by the TEM micrographs. These results suggest that bio-based and biodegradable PLA films filled with L, and particularly NL, can be employed as competitive and green alternatives in the food packaging industry.

## 1. Introduction

The significant environmental impact of petrochemicals, as well as the depletion of petroleum resources, calls for the rapid replacement of fossil-based materials with biodegradable ones manufactured from renewable resources. One of the most promising bio-based polymers is polylactic acid (PLA), an aliphatic polyester that can be obtained from renewable resources rich in starch or sugars, such as corn, potatoes, and sugarcane. Starch can be enzymatically hydrolyzed to produce sugars, and sugars can be fermented to produce lactic acid. [1,2]. PLA is mainly synthesized by the ring-opening polymerization of lactide or the direct condensation polymerization of lactic acid [3]. PLA is a biodegradable, biocompatible, non-toxic, and readily processible polymer that can compete with fossil-based counter plastics, contributing towards a circular economy [4,5]. Therefore, PLA is already used in industrial applications, such as biomedical [6], agriculture [3], food packaging [7,8], automotive [9], additive manufacturing [10], and printed electronics [11]. 

Nevertheless, PLA presents some drawbacks such as inherent brittleness (e.g., <10 Mpa tensile strength), low crystallization rate, quite slow biodegradation in the soil, moderate gas barrier properties, and low thermal stability [12]. To overcome these limitations and produce PLA-based materials with enhanced mechanical and physicochemical properties, scientific research has been directed towards the synthesis of composites using bio-based fillers such as lignin, cellulose, and their nano-counterparts [13,14]. These lignocellulosic materials are two of the most abundant organic resources in nature and account for approximately 50–75% of biomass [15]. 

Lignin, in particular, is a biopolymer derived from agro-industrial waste residues. The source and pre-treatment process of lignin determine its biological, morphological, and physicochemical characteristics [16]. Lignin has a three-dimensional randomly cross-linked heterogeneous structure, comprising a polyphenolic aromatic backbone and various surface functional groups, such as hydroxyl, carbonyl, and carboxylate groups [17]. Lignin has a high carbon content and shows good antioxidant, antimicrobial, flame retardant, and barrier properties [18]. Although lignin is highly resistant to chemical and biological degradation, it is slowly degraded by ligninolytic microbes [19]. Therefore, the integration of lignin with bio-based polymers can lead to renewable composites with improved properties [20,21]. 

To this end, commercially available raw lignin (L) has recently been investigated as a filler in PLA-based composites. Spiridon et al. [22,23] reported the synthesis of PLA-L composites with Organosolv or Lignoboost^®^ lignin at 7 and 15 wt% loading by compounding at 175 °C. On the one hand, the synthesized PLA–L composites showed a gradual increase in thermal stability, Young’s modulus, and impact strength with increasing lignin content. On the other hand, the elongation at break and tensile strength were significantly reduced. Fereira da Silva et al. [24] demonstrated that the addition of 5–15 wt% Kraft lignin to PLA by melt mixing at 200 °C increased the thermal stability, as well as biodegradability of the respective PLA-L composites. Importantly, the impact strength increased by 12% with the addition of Kraft lignin 5 wt% but decreased at higher loadings of 10 and 15 wt%. They noted that lignin was not a reinforcing agent but was added to promote biodegradation. Singla et al. [25] prepared PLA-L composites by compounding PLA with soda-lignin at 5–30 wt% loading. All the composites showed a gradual decrease (up to 30%) in tensile strength, elongation at break, and impact strength. Similar results were obtained by Triwulandari et al. [26] using lignin as a filler for PLA, polypropylene, and mixtures thereof. Li et al. [27] reported that manual mixing followed by compression molding at 200 °C under 5 Mpa pressure could only blend PLA and lignin at the macroscopic level, which in turn led to poor mechanical properties. 

Chaubey et al. [28] prepared PLA-L composites by co-polymerizing lactic acid with 2 wt% lignin in DMSO at 180 °C. Solvent casting in a poly(vinyl alcohol) aqueous solution was used to prepare PLA-L films that exhibited very good antimicrobial properties and a shorter biodegradability time in soil. Likewise, Buzarovska et al. used solvent casting in dioxane solvent to prepare 120 ± 10 μm thick PLA–L films with low molecular mass alkali lignin (1–10 wt%) [29]. The films showed weaker mechanical properties compared to neat PLA, even at 1 wt% lignin loading. By contrast, the water barrier properties increased up to 73% with increasing lignin content, which was attributed to the more hydrophilic character of the low molecular weight alkali lignin. In addition, PLA–L films showed excellent antimicrobial activity, reducing the viability of Gram-positive bacteria tenfold. Similar results were obtained by Cresnar et al. [30] and by Domenek et al. [31], who prepared PLA–L films by melt-mixing using Kraft and Soda lignin, respectively. Both studies also reported greater antioxidant activity with increasing lignin content. 

The above works indicated that the addition of different types of raw lignin as a bio-based filler to prepared PLA–L composite systems using various methods (co-polymerization, melt compounding, molding, solvent casting) did not significantly improve the mechanical properties compared to neat PLA. By contrast, the introduction of raw lignin enhanced the antimicrobial and antioxidant properties of PLA–L films. Overall, the mechanical performance was dictated by the compatibility between the PLA polymer matrix and the lignin filler, their interfacial adhesion, the particle size, and the dispersion of lignin in the PLA matrix [22,25,26]. Importantly, raw lignin contains a high number of large micron-sized particles with hydrophilic surface groups. This lowers the miscibility with hydrophobic PLA, which in turn leads to lignin particle aggregation and phase separation in the resulting composites. In addition, there are strong, although indirect effects, of lignin that are depicted in the crystallinity of the composites [32,33]. 

Efforts have been made to modify lignin with functional groups, to achieve better compatibility with polymer matrices [18]. Acetylation of lignin hydroxyl groups has been reported to improve the interaction with the hydrophobic ester groups of PLA, increase lignin dispersion, and therefore improve the mechanical performance of the respective composite materials [34,35,36,37]. Graft polymerization of PLA from lignin has also been demonstrated [38,39,40,41]. However, chemical modifications present many disadvantages for upscaling, such as the use of organic solvents, thermal treatment, increased cost of the final lignin, etc.

An alternative and more environmentally benign approach to prepare more homogeneous PLA-based composites with lignin involves the incorporation of lignin nanoparticles (nanolignin, NL). NL has a smaller and more uniform particle size, resulting in higher miscibility with PLA compared with microscale raw lignin [39,42,43,44,45]. To this end, Yang et al. [46] prepared two series of PLA–NL nanocomposite films using solvent casting or melt extrusion containing 1–3 wt% NL. Most importantly, aggregation of NL was observed in the solvent cast films, leading to poor mechanical properties. By contrast, a homogeneous dispersion of NL in the PLA matrix was obtained for the samples prepared by melt extrusion, which in turn resulted in the enhancement of the tensile strength, Young’s modulus, and elongation at break.

In this work, a comparative structure–properties investigation of PLA-based composites with micron-sized or nano-sized soda lignin as a filler, at four different loadings between 0.5 and 5 wt%, was performed. Notably, the composites were prepared by combining the two previously used methods of solvent casting and melt mixing, which in turn improved the dispersion of lignin in the PLA matrix. The prepared composites were pressed into films to investigate their physicochemical and morphological properties via differential scanning calorimetry (DSC), Fourier transform infrared spectroscopy (FTIR), X-ray diffraction (XRD), polarized light microscopy (PLM), and transmission electron microscopy (TEM). Regarding the thermal transitions, with an emphasis on glass transition and crystallization, we additionally performed a parallel study involving neat PLAs of various molecular weights, in order to disentangle the chain length effects from the direct effects of lignin. Such a work is not trivial in the literature. Finally, the effect of replacing micron-sized with nano-sized lignin on the mechanical and antioxidant properties of the films was investigated and is presented herein for the first time. 

## 2. Materials and Methods

### 2.1. Materials 

Biobased PLA (Ingeo™ Biopolymer 3052D, NatureWorks, Plymouth, MN, USA) of molar mass Mw~116,000 g/mol, with ~96% of L- and ~4% of D- forms was purchased from Plastika Kritis S.A. (Iraklion, Greece). Raw soda lignin (Protobind 1000) was purchased from Tanovis AG (Rüschlikon, Switzerland). Soda nanolignin was kindly provided by Creative nano PC (Athens, Greece). 2,2-diphenyl-1-picrylhydrazyl (DPPH, 95%) was purchased from Sigma-Aldrich (St. Louis, MO, USA). All materials were used as received. Analytical grade organic solvents were purchased by Sigma-Aldrich. 

### 2.2. Preparation of PLA-Lignin/Nanolignin Composites

#### 2.2.1. Masterbatch Synthesis via Solution Casting

PLA-L/NL masterbatches were initially prepared by solution casting. In summary, 2 g of PLA was dissolved in 20 mL of chloroform (10% *w*/*v*). Subsequently, the quantity of L/NL required for a final concentration of 0.5 wt%, 1 wt% 2.5, and 5 wt% in 10 g of PLA-L/NL composites was diluted in acetone (3% *w*/*v*) and was dispersed in a ultrasonication bath for 1 h, to achieve complete dissolution. The two different solutions were mixed under magnetic stirring, placed in Petri dishes, and left overnight for the solvents to evaporate. 

#### 2.2.2. Synthesis of PLA-Lignin/Nanolignin Composites via Melt Mixing of Masterbatch

The PLA and PLA-L/NL masterbatches were dried overnight under vacuum at 110 °C. To prepare the composites by melt mixing, the appropriate amount of each masterbatch and dried PLA were added to a melt- mixer with roller blades and a mixing head with a volumetric capacity of 11 cm^3^, operating at 190 °C and 30 rpm for 15 min. In total, eight composites, four containing Lignin and four containing Nanolignin, at different loadings of 0.5, 1, 2.5, and 5 wt% were prepared.

Within the scope of the calorimetric study, we also prepared a series of neat PLA samples of different [η], varying in the same range as those of PLA-L/NL compounds. These neat PLA samples were prepared by controlled hydrolysis of the initial PLA. Neat PLA was added to the batch reactor at 220 °C for 15, 30, and 60 min, to obtain the desired intrinsic viscosity values.

#### 2.2.3. Film Preparation 

The respective films were prepared by compression molding using an Otto Weber, Type PW 30 hydraulic press connected with an Omron E5AX Temperature Controller (Kyoto, Japan), at a temperature of 180 ± 5 °C and a pressure of 100 mbar. After melt pressing, the films were cooled rapidly at room temperature. Half of the prepared films were annealed overnight at 115 °C. All the films had a similar thickness of approximately 0.25 mm. 

### 2.3. Experimental Methods 

#### 2.3.1. Particle Size Distribution of Lignins

The average particle size of raw lignin and nanolignin was measured using dynamic light scattering (DLS) on a Litesizer 500 instrument (Anton Paar, Graz, Austria). The powders were dispersed in water at a concentration of 100 ppm via ultrasonication for 5 min prior to measurement. The hydrodynamic diameter was 2.38 μm (polydispersity index, PDI = 0.29) for raw lignin and 524 nm (PDI = 0.16) for nanolignin. Particle size distribution curves of both samples are presented in Figure S1. 

#### 2.3.2. Intrinsic Viscosity/Molecular Weight 

The intrinsic viscosity of the synthesized composites was measured with an Ubbelohde viscometer (Schott Gerate GMBH, Hofheim, Germany) at 25 °C, using chloroform as the solvent. Each sample was heated in the solvent mixture at 80 °C for 20 min, until complete dissolution. After being cooled, the solution was filtered through a disposable Teflon filter, to remove possible solid residues. Calculation of the intrinsic viscosity value of the polymer was performed by applying the Solomon–Cuita Equation (1) of a single point measurement:

(1) [η]=[2{tt0−ln(tt0)−1}]12c,

where *c* is the concentration of the solution, *t* is the flow time of the solution, and *t*_0_ is the flow time of the solvent. The experiment was performed three times, and the average value was estimated. 

The molecular weight values were calculated using the Mark–Houwink Equation (2) for PLA [1]:

(2) $$M_{\bar{n}}=2.21\times 10^{4\;}\times {\left[\eta \right]}^{0.77},$$

#### 2.3.3. Fourier Transform Infra-Red Spectroscopy (FTIR)

ATR-FTIR spectra were recorded on a Tension 27 FTIR spectrometer (Bruker, Billerica, MA, USA) equipped with a diamond ATR accessory at a spectral resolution of 4 cm^−1^ in the 4000–600 cm^−1^ range on the amorphous films. FTIR spectra were analyzed with the OPUS software (version 5.2, Bruker, Billerica, MA, USA). 

#### 2.3.4. Differential Scanning Calorimetry (DSC)

The thermal transitions of PLA, with focus on the glass transition and crystallization, were followed by differential scanning calorimetry (DSC). To that aim, we employed a TA Q200 series DSC instrument (TA Instruments, New Castle, DE, USA), calibrated with sapphires for heat capacity and indium for temperature and enthalpy. The measurements were performed in a high-purity nitrogen (99.9995%) atmosphere, on samples of ~7–8 mg in mass closed in Tzero aluminum pans and in the temperature range from −20 to 190 °C. Upon erasing the thermal history of each sample, two main protocols were employed, one involving cooling from the melt state at 10 °C/min and another involving a faster cooling at an average rate of ~100 °C/min. The two rates aimed at the manipulation of the polymer crystallization. The heating rate was fixed to 10 °C/min. More details are given below with the experimental results. 

#### 2.3.5. X-ray Diffraction (XRD)

XRD was employed to study the semi-crystalline structure of all samples at room temperature that had previously been melted and, subsequently, fully annealed (115 °C, overnight). The XRD spectra were recorded by means of a MiniFlex II XRD system (Rigaku Co., Tokyo, Japan), with Cu Ka radiation (0.154 nm), over the 2*θ* range from 5° to 50° with a scanning rate of 1°/min.

#### 2.3.6. Polarized Light Microscopy (PLM)

A polarizing light microscope (Nikon, Optiphot-2, Tokyo, Japan) equipped with a Linkam THMS 600 heating stage, a Linkam TP 91 control unit, and a Jenoptic ProgRes C10Plus camera with Jenoptik ProgRes CapturePro software was utilized for PLM observations. The samples were heated, melted, quenched, and captured during cold crystallization. 

#### 2.3.7. Transmission Electron Microscopy (TEM)

TEM images were obtained with an FEI Tecnai G2 20 microscope (FEI, Hillsboro, OR, USA) at the accelerating voltage of 200 kV. For the TEM sample preparation, PLA films were embedded in epoxy resin (Araldite CY212). After a 48 h curing, they were cut with ultramicrotome at 80 nm thickness (DiATOME 45° diamond knife, DiATOME ltd., Nidau, Switzerland). The thin sections floating on the knife’s water surface were deposited on carbon-coated grids and air-dried overnight.

#### 2.3.8. Tensile Testing

Tensile tests of the amorphous films were performed using an Instron 3344 dynamometer (Instron, Norwood, MA, USA), in accordance with ASTM D638, using a crosshead speed of 5 mm/min. Dumb-bell-shaped tensile test specimens (central portions 5 mm × 0.5 mm thick, 22 mm gauge length) were cut in a Wallace cutting press. At least five measurements were conducted for each sample, and the results of the respective stress–strain curves were analyzed and averaged to obtain the mean values of Young’s modulus, tensile strength, and elongation at break. The Young’s modulus was calculated from the slope of the linear part of the stress–strain curves after least squares linear fitting.

#### 2.3.9. Antioxidant Activity

The antioxidant activity of neat PLA, and PLA–L and PLA–NL composites, was evaluated by monitoring the reduction rate of the DPPH• radical in the antioxidant’s presence via UV-Vis spectroscopy. A 0.079 mM DPPH• solution in EtOH was prepared and stored in the dark for 16 h at room temperature. The 9 prepared films (1 neat PLA, 4 PLA-L, and 4 PLA-NL in 4 different loadings of L and NL, respectively) with the same dimensions (1 cm × 1 cm) were immersed in 3 mL of the DPPH•/EtOH solution at room temperature and kept in the dark. The composites’ antioxidant capacity was determined by measuring the absorption decay at 517 nm at regular time intervals. The residual DPPH content in the solution was calculated using Equation (3):

Residual DPPH content (%) = 100 — 100 (A_0_ — A_1_/A_0_), (3)

where A_0_ is the absorbance of the control sample and A_1_ is the absorbance in the presence of the films.

#### 2.3.10. Optical Properties 

The Diffuse Reflectance spectra of the PLA-based composites were measured using an Agilent Carry 60 spectrophotometer (Agilent Technologies, Santa Clara, CA, USA) equipped with a Harrick VideoBarrelino DRA fiber optic coupler (Pleasantville, NY, USA) between 200 and 800 nm. 

#### 2.3.11. Contact Angle 

Water contact angle measurements were performed using an optical tensiometer, One Attention (Biolin Scientific, Espoo, Finland). The sessile water droplet method was used to investigate the hydrophilicity of the PLA-based films due to the addition of L and NL. Measurements were performed in triplicate.

## 3. Results and Discussion 

### 3.1. Effect of L/NL on the Molecular Weight of the PLA Composites

**Figure 1:**
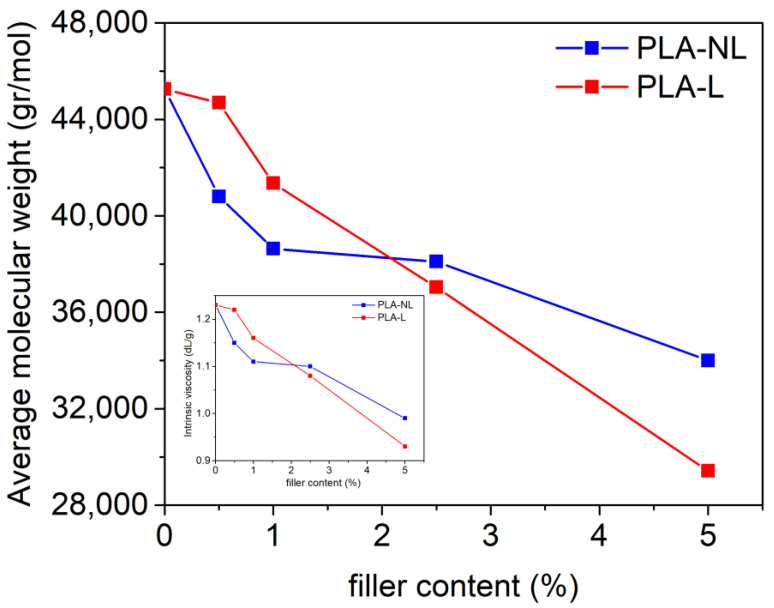
$M_{\bar{n}}$ and intrinsic viscosity (inset) of PLA–L (red) and PLA–NL (blue) composites with different filler contents.

The effect of L or NL addition on the molecular weight of the prepared composites was initially investigated. Figure 1 shows that the intrinsic viscosity and, subsequently, the molecular weight $(M_{\bar{n}}$) of the PLA–L and PLA–NL composites decreased with increasing filler content. As previously reported, this can be attributed to the lignin surface functional groups, such as hydroxyls and carboxylates, that tend to reduce the molecular weight of the PLA-based composites during melt mixing [31,47]. It should be noted, however, that the $M_{\bar{n}}$ decreased almost linearly with increasing filler content when raw lignin was used as the filler. As a result, $M_{\bar{n}}$ was reduced by 35% as compared to neat PLA for the PLA–L composite with the highest filler loading of 5 wt%. By contrast, a less steep reduction of $M_{\bar{n}}$ was observed in the case of NL filler, and $M_{\bar{n}}$ was decreased by 25% at 5 wt% NL loading.

### 3.2. Interactions between L/NL and PLA investigated by FTIR Spectroscopy

**Figure 2:**
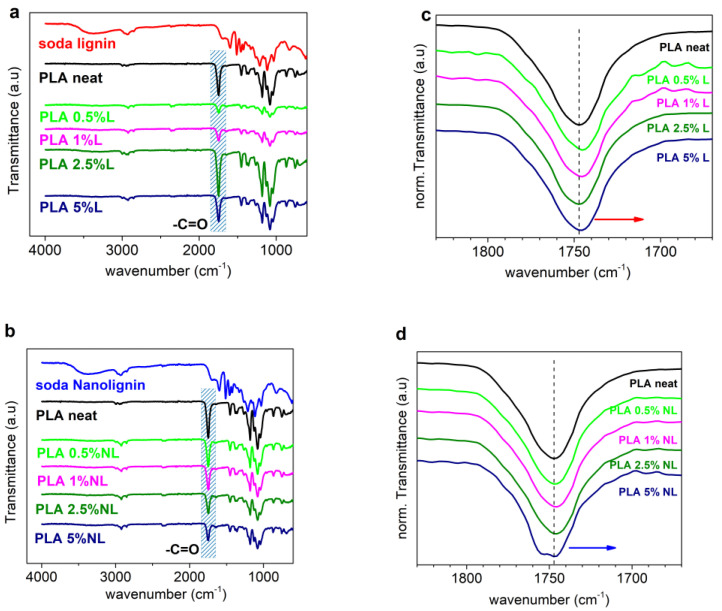
Comparison of the ATR−FTIR spectra of PLA and all the (**a**) PLA−L and (**b**) PLA−NL composites. Overlay of the corresponding (-C=O) peaks after intensity normalization for PLA−L (**c**) and PLA−NL composites (**d**). Red and blue arrows indicate the minor shift of composites spectra toward lower wavenumbers.

Figure 2a,b present the ATR-FTIR spectra of the starting materials (L, NL, and neat PLA) and all the synthesized composite films. First, L and NL have practically identical spectra (Figure S2). The band around 3400 cm^−1^ was assigned to the aromatic and aliphatic -OH surface groups. Transmittance bands in the region between 3000 and 2840 cm^−1^ were attributed to the stretching of the C–H bonds in the -OCH_3_ groups of lignin. In addition, the transmittance bands at 1600 cm^−1^, 1515 cm^−1^, and 1425 cm^−1^ were assigned to the typical aromatic ring vibrations of the phenylpropane skeleton [39,48].

For the neat PLA, the strong band at 1747 cm^−1^ was attributed to the -C=O stretching vibration of the ester groups. The bands at 2995 cm^−1^ and 1075 cm^−1^ were assigned to the stretching of the -CH_3_ and C-O bonds, respectively [49]. The hydroxyl surface groups of lignin have a strong tendency to form hydrogen bonds with the carbonyl group of PLA [25,27,29]. Obviously, we refer to free carbonyls (non-bound within other interactions). We, therefore, turned our attention to the characteristic -C=O stretching band of PLA at 1747 cm^−1^ (blue shaded in Figure 2a,b). In each spectrum, the peak intensity was normalized to (0, 1) (Figure 2c,d) [32,33]. 

For both series of composites, a minor shift toward lower wavenumbers was observed. This is indicative of interfacial interactions between the fillers and PLA [26,31]. Nevertheless, the changes in the peak position and shape were more pronounced in the case of the PLA–NL composites, culminating in the split of the band for 5 wt% NL loading, probably due to the formation of new bonds between the -OH groups of L/NL and the ester groups of PLA. Overall, the filler-polymer interactions were considerably stronger in the case of nanolignin. 

### 3.3. Differential Scanning Calorimetry Studies 

We now turn our attention to the thermal transitions, in particular, to the effects of lignin on the glass transition, crystallization, and melting of PLA. Prior to that, we note that, since the viscosity and molar mass dropped in the composites, this should have significantly affected the glass transition and crystallization [50,51,52] (both nucleation and crystallinity degree). Thus, for the sake of completeness, it was necessary to compare these composites with neat PLAs of similar [η]. To that aim, next to the initial PLA (of [η] = 1.24 dL/g), we prepared and studied additional neat PLA samples of lower [η] (1.14, 1.01 and 0.84 dL/g), which were prepared via controlled hydrolysis of our initial PLA [53].

**Figure 3:**
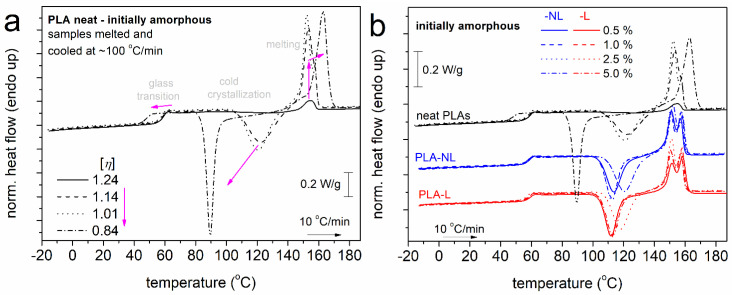
Comparative DSC heating traces for (**a**) neat PLAs of different [η] and (**b**) PLA and PLA−lignin composites, for all samples initially melted and cooled at a fast rate (initially amorphous). The recorded heat flow (in mW) has been normalized to each sample mass (in W/g). In (**a**), the added arrows mark the effects of the decrease in [η].

All samples were subjected to an initial heating up at 190 °C, i.e., well above melting, in order to erase the thermal history, any crystal memory, and evaporate any remaining solvents. Subsequently, the samples were cooled at 10 K/min. During that cooling (not shown), none of the systems exhibited a melt crystallization peak. However, during the subsequent heating, all samples exhibited cold crystallization and melting. This has been observed previously for the mentioned PLA and was explained in terms of the large supercooling [54] necessary for this combination of D-lactide and molar mass [55]. To further enhance the supercooling and, thus, the nucleation, we performed fast cooling at ~100 K/min, within the expected crystallization temperature range, for the initially melted samples. In Figure 3, we present the results of the subsequent heating for neat PLAs with different [η] values (Figure 3a) and PLA–L and PLA–NL composites (Figure 3b).

In all cases and in the order of increasing temperature, in Figure 3, the samples exhibit single glass transition steps in the range between 40 and 60 °C, cold crystallization exotherms between 70 and 140 °C, and complex melting endotherms between 140 and 170 °C. The results of Figure 3 were evaluated in terms of the characteristic temperatures for glass transition, *T*_g_, cold crystallization, *T*_cc_, and melting, *T*_m_, as well as in terms of the heat capacity change during glass transition, Δ*c*_p_, and crystallization/melting enthalpy changes, Δ*H*_cc_/Δ*H*_m_.

It is worth noting that the recorded glass transition steps and initiation of cold crystallization corresponded to fully amorphous, although nucleated, polymers. This suggests that any effects on the glass transition arose directly from either the chains’ length ([η]) or/and the presence of NL and L. The extreme cases are recorded in Figure 3b for the two PLAs of higher and lower [η], as expected. Therefore, it is essential to discuss the effects of lignin in terms of the similar or comparable [η] of the “matrix–PLA”.

**Figure 4:**
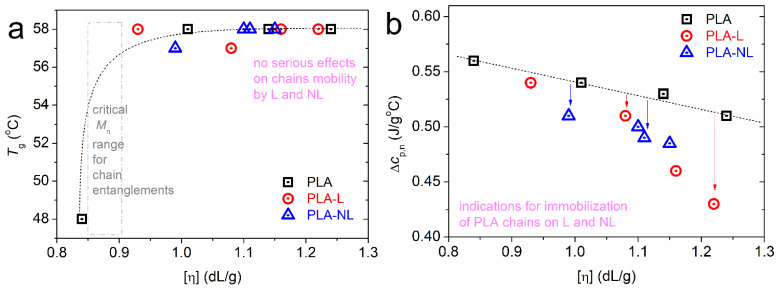
The [η] dependence on (**a**) glass transition temperature and (**b**) normalized heat capacity change. The lines connecting the experimental points were added to guide the eye.

In Figure 4a, we show the non-significant alternations in the *T*_g_ (57–58 °C) with the addition of lignin. In addition, none of our composites contained severely short chains, namely, below the molar mass threshold for entanglements [56]. Thus, we found a similar degree of chain–chain associations during glass transition, whereas, it seems that the mobile chain dynamics were not hindered by the presence of L and NL. 

On the other hand, the recording referring to the strength of glass transition in Figure 4b is interesting. Therein, the Δ*c*_p_ has been plotted with normalization to the PLA mass, Δ*c*_p,n_. It is shown that for a given [η] of the matrix, the Δ*c*_p,n_ dropped with the presence of lignin. Moreover, there was a stronger effect of NL compared to L. Since the polymers are amorphous, we could employ a widely adopted model to rationalize this systematic effect of lignin. According to Schick and coworkers [57,58], the suppression of Δ*c*_p_ in polymer nanocomposites originates from the formation of a “rigid amorphous” polymer fraction (i.e., the so-called RAF) around the nanofillers. RAF consists of immobilized polymer chains and does not contribute to the glass transition; thus, this is usually recorded as a missing part in Δ*c*_p_. This model has been confirmed in many cases of polymer composites in the literature [59,60,61,62,63,64]. For a fixed amount of filler, RAF increases when increasing the filler’s aspect ratio [61] and, equivalently, when decreasing the size [51,62,64,65,66]. Our results are in accordance to the latter, as the “smaller” NL particles imposed a larger reduction in Δ*c*_p,n_ than L. In addition, the results provide indications of the formation of PLA–lignin interactions [32]. Therefore, we can propose that the part missing from Δ*c*_p_ could be due to either special chain conformations and interactions formed in the composites or related to the crystallization nuclei.

**Figure 5:**
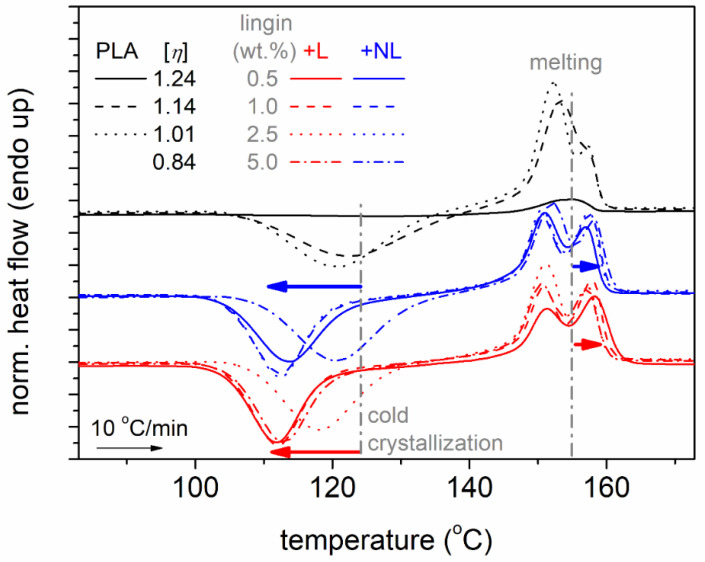
DSC heating traces for all samples, composites, and neat PLAs, described in a plot, focusing on the temperature regions of cold crystallization and melting. The added arrows mark the effects imposed by -NL and -L.

In the next section, we proceed with the direct effects of lignin on the crystallization. To facilitate the discussion, a focus on the higher temperature range of the DSC traces is shown in Figure 5. The most striking effects were recorded for cold crystallization, as can be discerned from the raw data. Cold crystallization is the result of incomplete crystallization during cooling; nevertheless, this involves stronger supercooling (more nucleation) [50] compared to melt-crystallization. 

**Figure 6:**
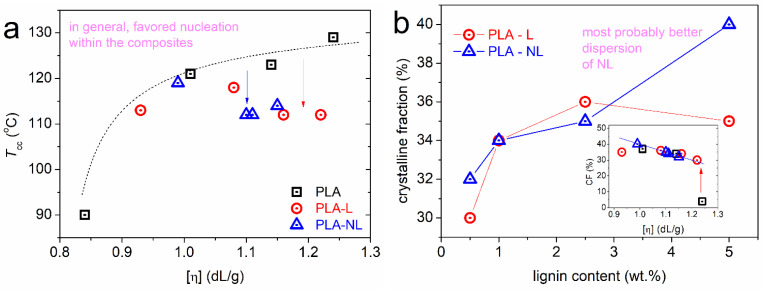
(**a**) The [η] dependence on the cold crystallization temperature for all samples. (**b**) The lignin loading dependence on the crystalline fraction, CF, estimated from cold crystallization. The inset to (**b**) shows the same data for CF as a function of the [η] of the matrix.

In all cases, the cold crystallization of the composites migrated toward lower temperatures. The data were further evaluated and are shown in Figure 6a,b, in terms of the characteristic peak temperature, *T*_cc_, and crystalline fraction. The crystalline fraction, CF, was estimated from Δ*H*_cc_ using Equation (4) where Δ*H*_100%_ is the enthalpy of 100% crystalline PLA taken as equal to 93 J/g according to Fischer et al. [67].

(4) $$CF=\Delta H_{cc}/\Delta H_{100\%},\;$$

We must report, for the sake of completeness, that more recently, larger values for Δ*H*_100%_ of PLA have been estimated. For example, [65] reported 107 and 143 J/g for the so-called *α*- and *α′* crystal -forms, respectively; whereas, Androsch and coworkers [68] reported a value of 104.5 ± 6 J/g for poly(L-lactic acid).

In Figure 6a, *T*_cc_ is lower compared to the unfilled PLA for a given [η]. This indicates that, for similar mobilities and lengths of the polymer chains, the presence of L and NL clearly facilitated nucleation. In Figure 6b, when considering only the lignin content, the CF seems to have been increased by lignin (from 30% to 40%), with the role of NL being more systematic compared to L. Nevertheless, when also taking into account the change in the [η] of the matrix, the mentioned CF favoring is actually artificial. This is expected, as the development rate of spherulites involves lamellae packings and these are connected to the chains’ fragility and length [66]. Combined with the results on crystallization, i.e., more nuclei in the composites, although a similar CF, we expected significant alternations in the composites’ semicrystalline morphology; namely, more spherulites, but smaller in size [50]. These points could be further illuminated by more direct measurements, for example, involving polarized light microscopy [32] (see below Section 3.5).

**Figure 7:**
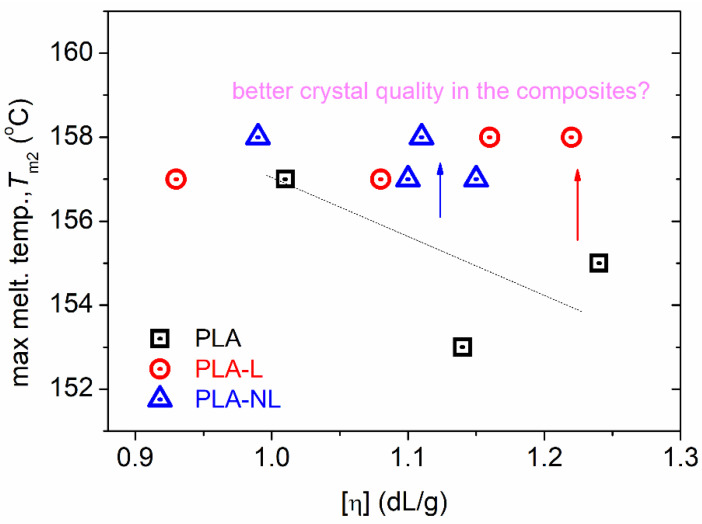
The [η] dependence on higher melting temperature, *T*_m2_, for all samples.

Finally, it should be noted that the melting was recorded within all samples as complex peaks at relatively low temperatures. This is the expected situation for cold crystallized PLA in combination with relatively low [η] [51,65,66] and is usually indicative of the formation of *α′* crystal forms. Within the complex melting region, the melting of low-quality or metastable crystals takes place, along with partial recrystallizations, and, finally, overall melting [50,51,69]. Thus, in Figure 7, we show the effects recorded with the highest melting temperature, *T*_m2_, as the optimum representative of the final crystals’ melting. Comparing again with neat PLAs of similar [η], the *T*_m2_ was recorded as elevated in the presence of lignin. This effect could suggest a better crystal quality, e.g., more dense crystals/lamellae packing.

### 3.4. X-ray Diffraction

**Figure 8:**
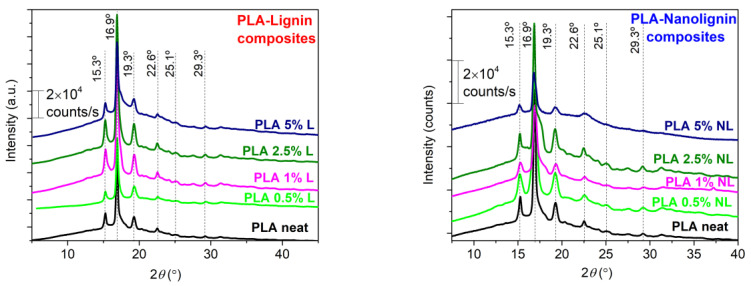
XRD results are shown comparatively for neat PLA and PLA–Lignin/Nanolignin composites that underwent isothermal crystallization annealing at 115 °C overnight.

XRD patterns of all composite films are shown in Figure 8, to evaluate the effect of L and NL on the crystal organization of PLA. The diffraction peaks at 16.9° and 19.3° are attributed to the more ordered α-crystal form, while the peaks at 15.3° and 22.6° are assigned to the more disordered α′-crystal form of PLA. The composites appear to crystallize in both forms, as reported for an annealing temperature between 100 and 120 °C [70]. The coexistence of both crystal forms corroborates the DSC measurements, where a double melting peak was observed. As proposed by Tabi et al. [71], the first melting peak may originate from the simultaneous α crystal melting, produced upon annealing, and the α′ to α recrystallization; while the second melting peak signifies the melting of the α crystal form produced from the α′ to α recrystallization process. Although it is hard to precisely define the crystal content of composites [3,29], the synthesized PLA-L/NL composites present a similar profile to neat PLA. This suggests that the type (α, α′ crystal forms) and the quality of PLA crystals is preserved in the composites, independently from the particle size and the content of the lignin filler.

### 3.5. Polarized Light Microscopy (PLM)

**Figure 9:**
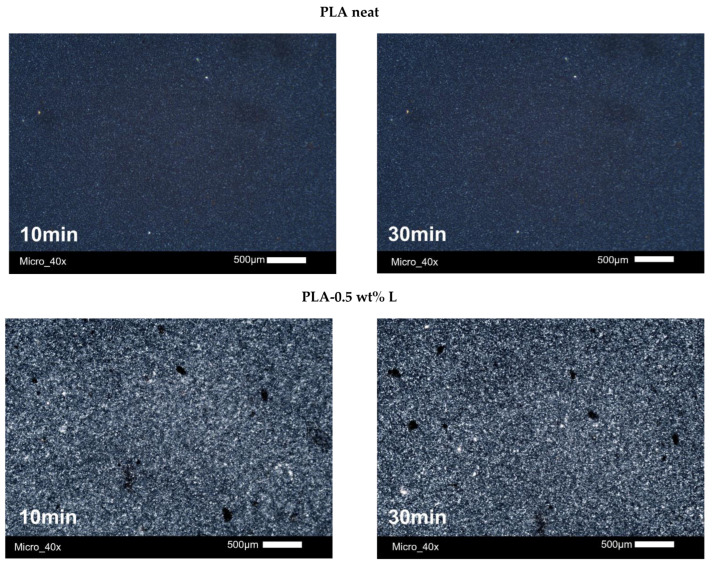

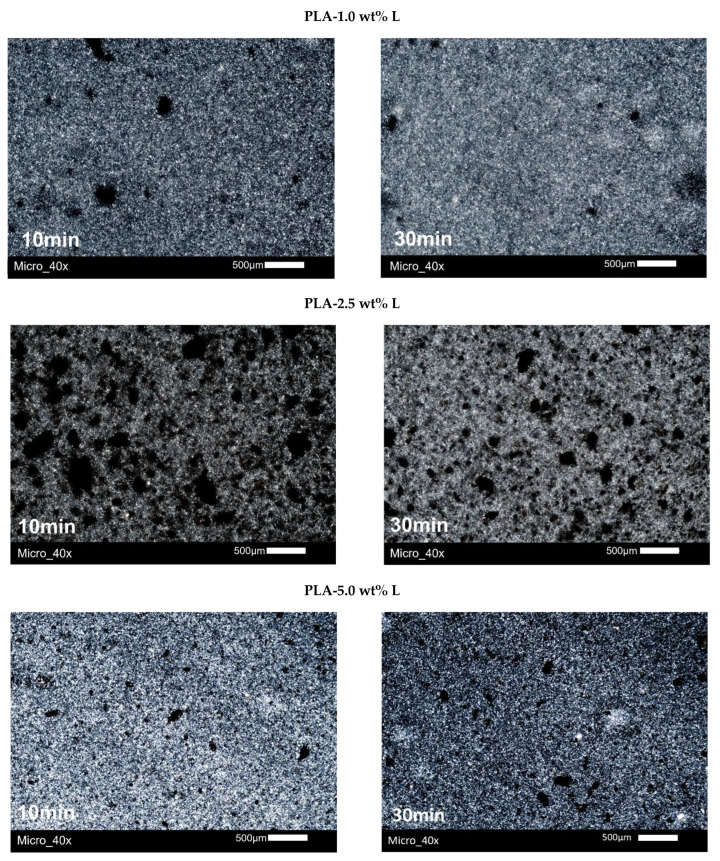
Microphotographs of neat PLA and PLA–lignin composites after annealing for 10 and 30 min using PLM.

**Figure 10:**
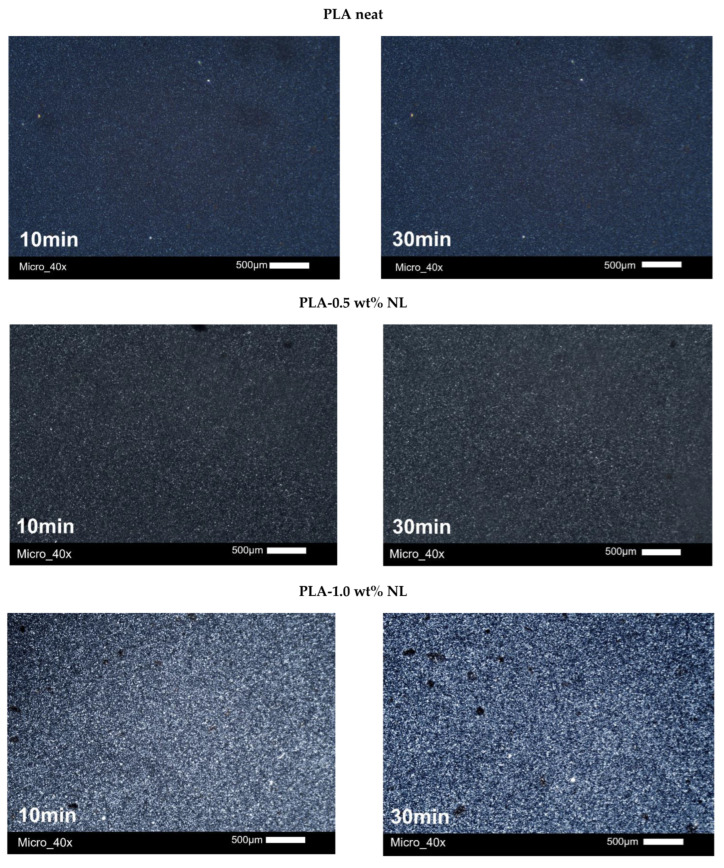

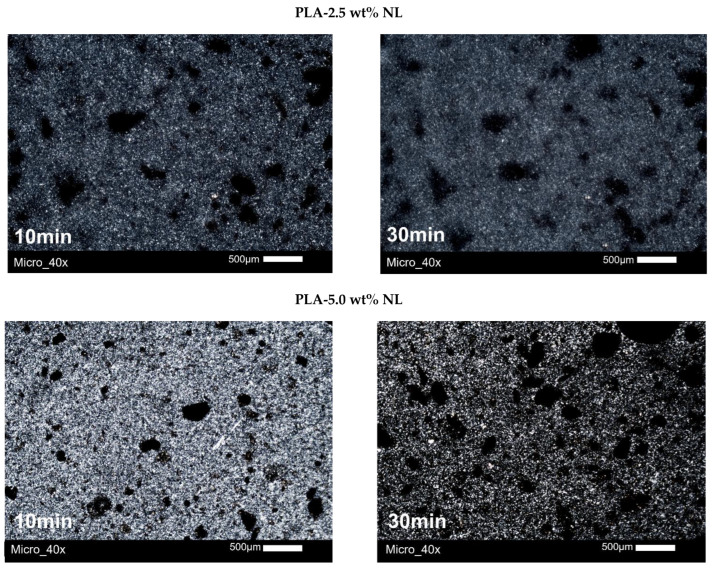
Microphotographs of neat PLA and PLA–nanolignin composites after annealing for 10 and 30 min using PLM.

The morphology of the PLA crystallites during the crystallization from the glass (cold crystallization) was investigated using PLM. All the samples were heated up to 185 °C and then quenched to room temperature. The quenched materials were then heated with a heating rate of 5 °C/min at 110 °C, a temperature at which all samples exhibited the phenomenon of cold crystallization [72]. The microphotographs of neat PLA, and PLA–L and PLA–NL composites, are presented in Figure 9 and Figure 10. As can be observed, the size of the crystals progressively increased and their number also increased with time for both series of composites, which can be attributed to the effect of the additive as a nucleation agent. These observations supplement the results obtained from the DSC measurements. 

The results of PLM revealed tremendous changes with respect to the semicrystalline morphology. By the latter term, we refer to the numbers, sizes, and distribution of polymer crystals throughout the sample volume. The addition of L and NL systematically led to larger crystallites, appearing as brighter spots in the PLM, when compared with neat PLA. This suggests a denser crystal structure (thicker lamellae packing) in the composites. This effect correlates well with the increase mentioned above in *T*_m2_ for the composites. Unfortunately, the optical resolution does not allow further distinguishing the effects arising from the different types (size) of fillers.

### 3.6. Dispersion of L/NL in PLA Composites 

**Figure 11:**
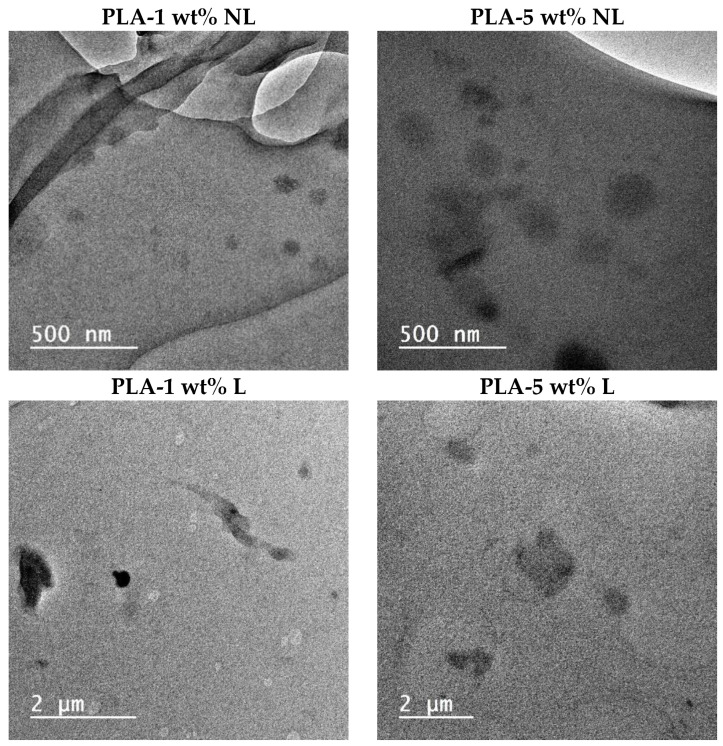
TEM micrographs of PLA–L/NL composites containing two different (1 and 5 wt%) filler contents.

The PLA-L/NL distribution was also investigated using TEM. From the TEM micrographs (Figure 11), the L and NL distribution in the PLA matrix was sufficient with the lower content, whereas some aggregation was observed at higher NL/L contents. In the case of PLA-1 wt% NL, sizes of lignin particles ranging from 30–40 nm to 100–120 nm are visible; while when increasing the NL content to 5 wt%, sizes ranging from 50 to 200 nm can be seen. From these micrographs, it is evident that the nanolignin retained its size during melt mixing with PLA. Comparing with the PLA–L composites, the lignin particle size at 1 wt% L content was distributed in the area of 300–1300 nm; while for 5 wt% L, the sizes were much larger, being 300–1800 nm. Therefore, the NL was dispersed in the nanosize range, even at the highest content (5 wt%); while L was dispersed in much higher sizes, mainly in the range of μm, which is in agreement with the scanning electron micrographs (Figure S5). This finer dispersion of NL could explain the slightly stronger interfacial interactions with PLA that were found by thermal analysis.

### 3.7. Mechanical Properties 

**Figure 12:**
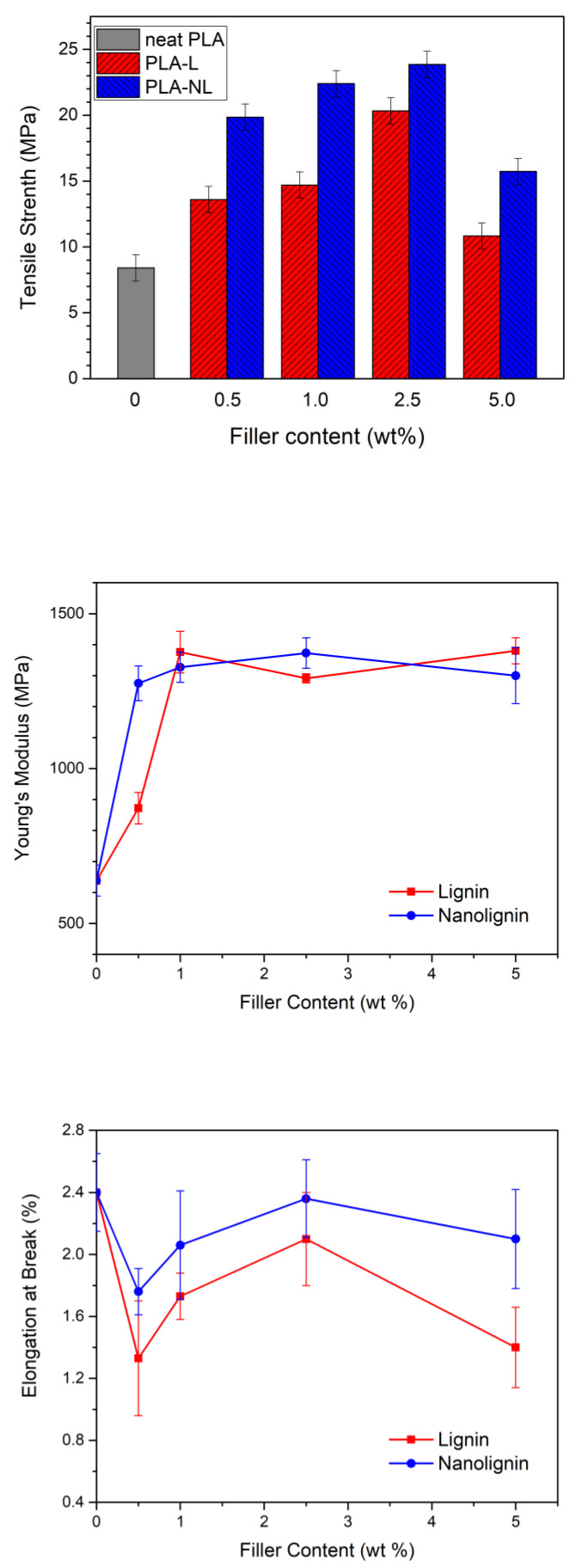
Mechanical properties of the PLA and PLA–Lignin/Nanolignin composites.

The mechanical properties of the composites are essential for understanding their ability to maintain their structure under mechanical stress, related to various applications [46]. In this work, the tensile strength, Young’s modulus, and elongation at break values of the eight different composite films and neat PLA (Figure 12) were obtained from stress–strain curves (Figures S3 and S4). The amorphous neat PLA presented weak mechanical properties, with characteristic values of tensile strength, elongation at break, and Young’s modulus of 8.41 MPa, 2.4%, and 638 MPa, respectively. The tensile strength increased by increasing the L/NL content between 0.5 and 2.5 wt%, but then decreased for the composites containing 5 wt% L/NL loading. A similar trend, where the tensile strength was reduced at lignin loadings greater than 5 wt%, was previously reported for other PLA–L composites utilizing raw unmodified lignins [31,34]. Nevertheless, all the PLA–L composites showed a higher tensile strength compared with neat PLA. Moreover, the addition of NL resulted in a higher tensile strength compared with L at the same loading, probably due to better the dispersion of NL in the PLA matrix. As a result, the maximum value of tensile strength was achieved for 2.5 wt% NL (23.86 MPa), a three-fold increase compared with neat PLA. These differences were likely due to the finer dispersion of NL in PLA matrix, as was verified in the TEM micrographs. 

The Young’s modulus showed a two-fold increase with NL addition, even at very low loadings of 0.5 wt%, compared to neat PLA, within experimental error. Specifically, the Young’s modulus increased from 638 ± 50 MPa for neat PLA to approximately 1300 ± 90 MPa for all PLA–NL composites. Similar values were observed for the PLA–L composites, albeit at L loadings greater than 1 wt%. The maximum values of 1380 MPa and 1373 MPa were obtained for 5 wt% L and 2.5 wt% NL, respectively. Sun et al. [41] suggested that both tensile strength and Young’s modulus increase with the incorporation of lignin, due to the rigidity of the lignin’s structure, which consists of highly concentrated benzene rings, as well as due to stereocomplexation, which promotes the load transfer from PLA to lignin and facilitates lignin dispersion in the PLA matrix [41]. 

Finally, the elongation at break was initially reduced with an increasing filler content, and all composites became more brittle compared to neat PLA. Notably, the elongation at break did not follow a specific trend with increasing filler content, and therefore the effect of L/NL addition could not be clearly identified. The reduction of the elongation at break was attributed to the brittle nature and the low molecular weight of the PLA, as well as to the rigidity of the lignin particles [22,26,47]. 

### 3.8. Antioxidant Properties of PLA–Lignin/Nanolignin Composites

**Figure 13:**
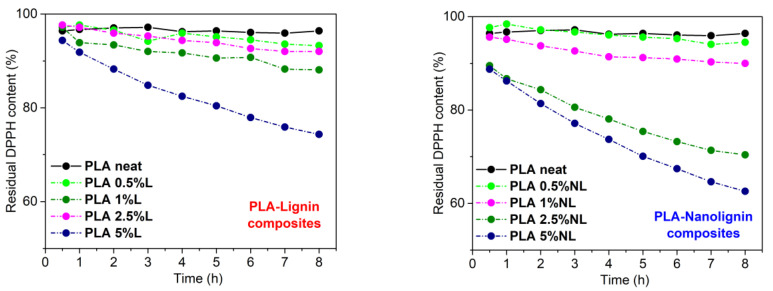
Reaction kinetics of the free radical DPPH during immersion of PLA–Lignin (**left**) and PLA-Nanolignin (**right**) films in ethanol solution.

The antioxidant ability of lignin stems from the presence of phenolic hydroxyl groups [73]. In this study, the radical scavenging activity of the composites was evaluated by monitoring the reduction of the DPPH radical (DPPH•). Figure 13 shows the residual DPPH content over time for different PLA–L (left) and PLA–NL (right) composites immersed in a DPPH/Ethanol solution. Importantly, the neat PLA sample showed negligible activity. By contrast, the addition of either L or NL in the PLA matrix enhanced the antioxidant activity, as could be deduced from the higher reduction rate of DPPH• over time. The composites with higher L/NL content showed better antioxidant activity, in agreement with the literature [30,43,74]. In the case of the PLA–L composites, the PLA–L composite with 5 wt% L loading yielded 74% residual DPPH content reduction after 8 h, while the PLA–L composites with lower L loadings (0.5–2.5 wt%) showed poor antioxidant activity. Concerning the PLA-NL composites, films with 2.5 and 5 wt% NL contents afforded 70% and 62% residual DPPH contents after 8 h, respectively. 

**Figure 14:**
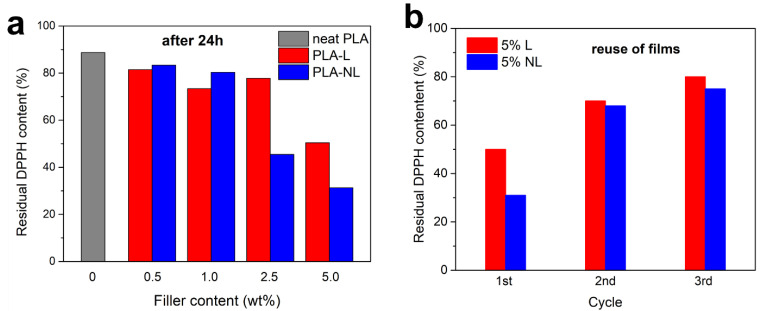
Residual DPPH content of composites after 24 h incubation (**a**). Reuse of the PLA–L/NL films with 5 wt% filler content (**b**).

Figure 14a shows the residual DPPH content after extending the immersion time of the PLA-L/NL to 24 h. Notably, the neat PLA film afforded only a 89% residual DPPH content in 24 h. All the composites investigated herein showed a higher antioxidant activity, which increased with an increasing filler content. The highest activity was observed for the PLA–NL films with 2.5 and 5 wt% NL loading, yielding 46% and 31% residual DPPH content in 24 h. This was accompanied by a gradual change in color of the respective EtOH solutions, from purple to light yellow (Figure S6). Reuse of the 5 wt% films showed a gradual decrease of antioxidant activity and thereby an increase of residual DPPH content from 31% to 68% to 75% for PLA–NL, and from 50% to 70% to 80% for PLA–L, after three consecutive 24 h cycles (Figure 14b). The stronger antioxidant capacity of the PLA–NL composites can be attributed to the larger specific surface area and the better dispersion of NL in the PLA-matrix [73].

### 3.9. Optical Properties of PLA and PLA–Lignin/Nanolignin Composites 

**Figure 15:**
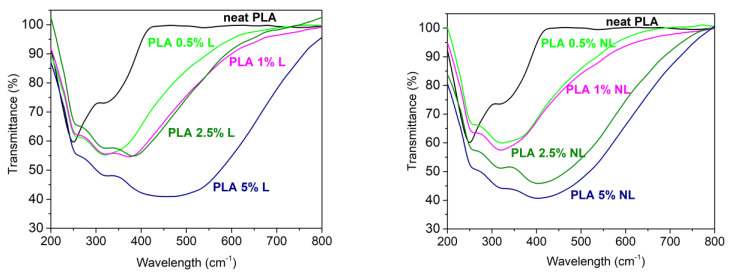
Optical properties of the neat PLA and PLA−Lignin (**left**) and PLA−Nanolignin (**right**) composites.

**Figure 16:**
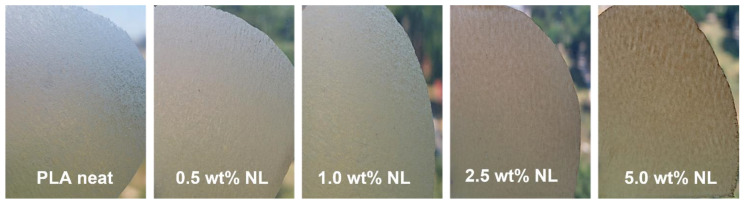
PLA–nanolignin films with different NL content.

The UV-Vis barrier properties of the PLA–L/NL composites synthesized herein were also investigated. UV-Vis barrier properties are crucial for PLA-based films, especially for food packaging applications, since UV radiation can cause degradation of plastic composites. The surface functional groups of lignin such as phenols, ketones, and chromophores can improve the UV absorbance of lignin-containing composites [37]. Figure 15 shows the UV-Vis transmittance of the films between 200–800 nm. The neat PLA film was practically transparent in the visible region and started to absorb below 400 nm. By contrast, all the composite films strongly absorbed in the visible region of the spectrum. In addition, the UV absorption was higher for all the composites and was filler concentration dependent. The better UV-Vis blocking capacity of the PLA–L/NL composites originated from the dark (yellow to brown) color, due to the chromophoric nature of lignin [28,40,75]. However, in the case of NL, the composites presented a higher transmittance compared to the lignin-based composites in the same concentration. According to the literature, the reduction in particle size of lignin particles can enhance the transmission of a film in visible light [36,38]. This also affected the visual appearance of the PLA-NL films; while, as can be seen from Figure 16, there was a macroscopically uniform distribution of nanolignin in the PLA matrix, without any visible particles or aggregates.

### 3.10. Contact Angle 

**Figure 17:**
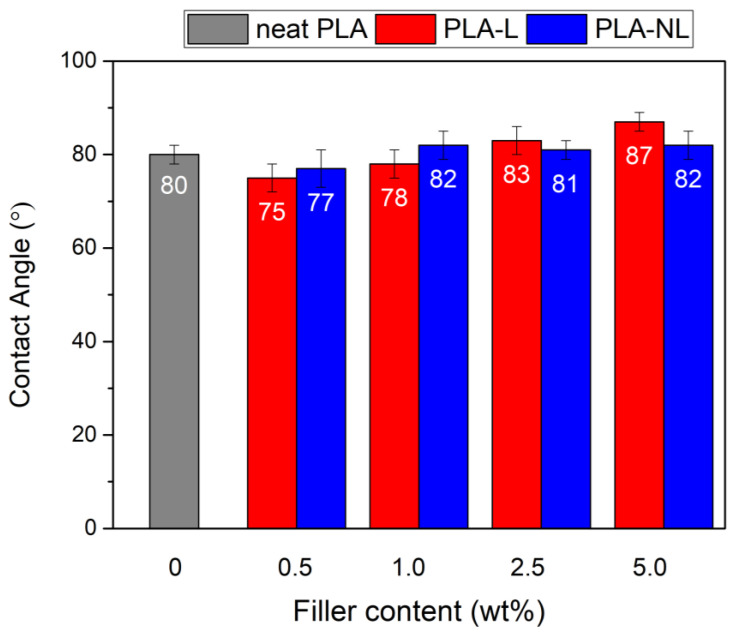
Contact angle of neat PLA and PLA–Lignin/Nanolignin composites.

Contact angle measurements of the PLA and PLA–L/NL composites were performed, to investigate the change in the hydrophobicity of the prepared films. Figure 17 shows that the addition of lignin slightly improved the hydrophobicity of the PLA–L/NL films for loadings greater than 1 wt%. The filler effect on the hydrophobicity was clear in the case of the PLA–L composites, where water contact angle increased with increasing L content, from 75° to 83° For the PLA–NL nanocomposites, the contact angle was stable at 81–82° and was practically independent of the NL concentration.

## 4. Conclusions 

In this study, unmodified lignin in micro- and nano-scale was used for the preparation of PLA biocomposites via combining solvent casting and melt mixing. Initially, the effect of fillers on the crystallization and semicrystalline morphology of the composites was investigated. The glass transition temperature of the composites was found to be unaffected; however, the corresponding heat capacity was reduced. When the change in molar mass in the composites was considered, the fillers were proven to play a facilitating role in the nucleation and resulted in a denser crystal structure in the composites. The FTIR and XRD results indicated non-negligible interactions between the lignin filler and the PLA matrix, especially in the case of NL. This was depicted in the tensile strength of the synthesized composites, which was significantly increased compared to neat PLA. Moreover, the tensile strength was consistently higher for the PLA–NL nanocomposites compared to their PLA–L counterparts at the same filler contents. Notably, the PLA–NL nanocomposite with 2.5 wt% NL showed a three-fold enhancement of tensile strength compared to the neat PLA. The Young’s modulus of the PLA–L/NL biocomposites was also increased, reaching maximum values of 1380 MPa and 1373 MPa for 5 wt% L and 2.5 wt% NL, respectively, compared to 638 ± 50 MPa for neat PLA. By contrast, the elongation at break of the composites decreased with the addition of both fillers. As revealed in the TEM micrographs, NL was dispersed in the PLA matrix in nanoscale range, while the L was mainly in μm sizes. The multifunctional effect of the L/NL integration with PLA was also demonstrated by evaluating the antioxidant capacity of the composites with a DPPH assay. The addition of L/NL in PLA resulted in an increase of the antioxidant capacity with increasing filler content. As above, the PLA–NL composites constantly outperformed their PLA–L counterparts at the same filler contents. For instance, the residual DDPH content for PLA-5 wt% L and PLA-5 wt% NL composites was 50% and 30% after 24 h, respectively. Finally, the chromophoric nature of lignin provided a UV blocking ability to the PLA-based films, which was proportional to the filler content. Overall, it was observed that the addition of lignin, and especially NL, which showed a better performance in all characterization studies, to PLA could be an alternative method for producing greener multifunctional PLA-based nanocomposites with added value for food packaging and other applications.

## Data Availability

All the data of this study is included in the manuscript and supplementary material.

## Supplementary Materials

The following supporting information can be downloaded at: https://www.mdpi.com/article/10.3390/polym14235274/s1. Figure S1: Particle size distribution curve of raw soda lignin and nanolignin corresponding to hydrodynamic diameter of 2.38 μm (polydispersity index, PDI = 0.29) and 524 nm (PDI = 0.161), respectively. Figure S2: FTIR spectra of soda lignin and nanolignin. Figure S3: Tensile stress-strain curves of PLA-L composites. Figure S4: Tensile stress-strain curves of PLA-NL composites. Figure S5: SEM images of PLA-L/NL composites containing different L/NL content. Figure S6: Qualitative antioxidant capacity of composites—Photographs of DPPH/ethanol solution for different PLA-Lignin (a) and PLA-Nanolignin (b) composites after 24 h exposure to PLA-L/NL films.

## References

[1] Garlotta D. (2001). A literature review of Poly(lactic acid). J. Polym. Environ..

[2] Saini P., Arora M., Ravi Kumar M.N.V. (2016). Poly(lactic acid) blends in biomedical applications. Adv. Drug Deliv. Rev..

[3] Taib N.-A.A.B., Rahman R., Huda D., Kuok K.K., Hamdan S., Bin Bakri M.K., Bin Julaihi M.R.M., Khan A. (2022). A review on poly lactic acid (PLA) as a biodegradable polymer. Polym. Bull..

[4] McKeown P., Jones M.D. (2020). The chemical recycling of PLA: A review. Sustain. Chem..

[5] Post W., Susa A., Blaauw R., Molenveld K., Knoop R.J. (2020). A review on the potential and limitations of recyclable thermosets for structural applications. Polym. Rev..

[6] Ebrahimi F., Ramezani Dana H. (2022). Poly lactic acid (PLA) polymers: From properties to biomedical applications. Int. J. Polym. Mater. Polym. Biomater..

[7] Balla E., Daniilidis V., Karlioti G., Kalamas T., Stefanidou M., Bikiaris N.D., Vlachopoulos A., Koumentakou I., Bikiaris D.N. (2021). Poly(lactic Acid): A versatile biobased polymer for the future with multifunctional properties—From monomer synthesis, polymerization techniques and molecular weight increase to PLA applications. Polymers.

[8] Psochia E., Papadopoulos L., Gkiliopoulos D., Francone A., Grigora M.-E., Tzetzis D., de Castro J., Neves N., Triantafyllidis K., Torres C. (2021). Bottom-up development of nanoimprinted plla composite films with enhanced antibacterial properties for smart packaging applications. Macromol.

[9] Bouzouita A., Notta-Cuvier D., Raquez J.-M., Lauro F., Dubois P., Di Lorenzo M.L., Androsch R. (2017). Poly(lactic acid)-Based Materials for Automotive Applications. Industrial Applications of Poly(Lactic Acid).

[10] Arockiam A.J., Subramanian K., Padmanabhan R., Selvaraj R., Bagal D.K., Rajesh S. (2022). A review on PLA with different fillers used as a filament in 3D printing. Mater. Today Proc..

[11] Atreya M., Dikshit K., Marinick G., Nielson J., Bruns C., Whiting G.L. (2020). Poly(lactic acid)-based ink for biodegradable printed electronics with conductivity enhanced through solvent aging. ACS Appl. Mater. Interfaces.

[12] Zhai S., Liu Q., Zhao Y., Sun H., Yang B., Weng Y. (2021). A Review: Research Progress in Modification of Poly(lactic Acid) by Lignin and Cellulose. Polymers.

[13] Saeidlou S., Huneault M.A., Li H., Park C.B. (2012). Poly(lactic acid) crystallization. Prog. Polym. Sci..

[14] John M.J., Lefatle M.C., Sithole B. (2022). Lignin fractionation and conversion to bio-based functional products. Sustain. Chem. Pharm..

[15] Shahzadi T., Mehmood S., Irshad M., Anwar Z., Afroz A., Zeeshan N., Rashid U., Sughra K. (2014). Advances in lignocellulosic biotechnology: A brief review on lignocellulosic biomass and cellulases. Adv. Biosci. Biotechnol..

[16] Li T., Takkellapati S. (2018). The current and emerging sources of technical lignins and their applications: Sources of Technical Lignins. Biofuels Bioprod. Biorefin..

[17] Katahira R., Elder T.J., Beckham G.T., Beckham G.T. (2018). Chapter 1. A Brief Introduction to Lignin Structure. Energy and Environment Series.

[18] Puglia D. (2022). Micro and Nanolignin in Aqueous Dispersions and Polymers: Interactions, Properties, and Applications.

[19] Ruiz-Dueñas F.J., Martínez Á.T. (2009). Microbial degradation of lignin: How a bulky recalcitrant polymer is efficiently recycled in nature and how we can take advantage of this: Action mechanisms of ligninolytic peroxidases. Microb. Biotechnol..

[20] Collins M.N., Nechifor M., Tanasă F., Zănoagă M., McLoughlin A., Stróżyk M.A., Culebras M., Teacă C.-A. (2019). Valorization of lignin in polymer and composite systems for advanced engineering applications—A review. Int. J. Biol. Macromol..

[21] Ridho M.R., Agustiany E.A., Rahmi Dn M., Madyaratri E.W., Ghozali M., Restu W.K., Falah F., Rahandi Lubis M.A., Syamani F.A., Nurhamiyah Y. (2022). Lignin as Green Filler in Polymer Composites: Development Methods, Characteristics, and Potential Applications. Adv. Mater. Sci. Eng..

[22] Spiridon I., Leluk K., Resmerita A.M., Darie R.N. (2015). Evaluation of PLA–lignin bioplastics properties before and after accelerated weathering. Compos. Part B Eng..

[23] Spiridon I., Tanase C.E. (2018). Design, characterization and preliminary biological evaluation of new lignin-PLA biocomposites. Int. J. Biol. Macromol..

[24] da Silva T.F., Menezes F., Montagna L.S., Lemes A.P., Passador F.R. (2019). Effect of lignin as accelerator of the biodegradation process of poly(lactic acid)/lignin composites. Mater. Sci. Eng. B.

[25] Kumar Singla R., Maiti S.N., Ghosh A.K. (2016). Crystallization, Morphological, and Mechanical Response of Poly(Lactic Acid)/Lignin-Based Biodegradable Composites. Polym.-Plast. Technol. Eng..

[26] Triwulandari E., Ghozali M., Sondari D., Septiyanti M., Sampora Y., Meliana Y., Fahmiati S., Restu W.K., Haryono A. (2019). Effect of lignin on mechanical, biodegradability, morphology, and thermal properties of polypropylene/polylactic acid/lignin biocomposite. Plast. Rubber Compos..

[27] Li J., He Y., Inoue Y. (2003). Thermal and mechanical properties of biodegradable blends of poly (L-lactic acid) and lignin. Polym. Int..

[28] Chaubey A., Aadil K.R., Jha H. (2021). Synthesis and characterization of lignin-poly lactic acid film as active food packaging material. Mater. Technol..

[29] Bužarovska A., Blazevska-Gilev J., Pérez-Martnez B.T., Balahura L.R., Pircalabioru G.G., Dinescu S., Costache M. (2021). Poly(l-lactic acid)/alkali lignin composites: Properties, biocompatibility, cytotoxicity and antimicrobial behavior. J. Mater. Sci..

[30] Črešnar K.P., Zamboulis A., Bikiaris D.N., Aulova A., Zemljič L.F. (2022). Kraft Lignin/Tannin as a Potential Accelerator of Antioxidant and Antibacterial Properties in an Active Thermoplastic Polyester-Based Multifunctional Material. Polymers.

[31] Domenek S., Louaifi A., Guinault A., Baumberger S. (2013). Potential of Lignins as Antioxidant Additive in Active Biodegradable Packaging Materials. J. Polym. Environ..

[32] Črešnar K.P., Klonos P.A., Zamboulis A., Terzopoulou Z., Xanthopoulou E., Papadopoulos L., Kyritsis A., Kuzmič K., Zemljič L.F., Bikiaris D.N. (2021). Structure-Properties relationships in renewable composites based on polylactide filled with Tannin and Kraft Lignin-Crystallization and molecular mobility. Thermochim. Acta.

[33] Terzopoulou Z., Klonos P.A., Kyritsis A., Tziolas A., Avgeropoulos A., Papageorgiou G.Z., Bikiaris D.N. (2019). Interfacial interactions, crystallization and molecular mobility in nanocomposites of Poly(lactic acid) filled with new hybrid inclusions based on graphene oxide and silica nanoparticles. Polymer.

[34] Gordobil O., Egüés I., Llano-Ponte R., Labidi J. (2014). Physicochemical properties of PLA lignin blends. Polym. Degrad. Stab..

[35] Gordobil O., Delucis R., Egüés I., Labidi J. (2015). Kraft lignin as filler in PLA to improve ductility and thermal properties. Ind. Crop. Prod..

[36] Kim Y., Suhr J., Seo H.-W., Sun H., Kim S., Park I.-K., Kim S.-H., Lee Y., Kim K.-J., Nam J.-D. (2017). All biomass and UV protective composite composed of compatibilized lignin and Poly(lactic-acid). Sci. Rep..

[37] Cavallo E., McPhee D.J., Luzi F., Dominici F., Cerrutti P., Bernal C., Foresti M.L., Torre L., Puglia D. (2020). UV Protective, antioxidant, antibacterial and compostable polylactic acid composites containing pristine and chemically modified lignin nanoparticles. Molecules.

[38] Chung Y.-L., Olsson J.V., Li R.J., Frank C.W., Waymouth R.M., Billington S.L., Sattely E.S. (2013). A renewable lignin–lactide copolymer and application in biobased composites. ACS Sustain. Chem. Eng..

[39] Yang W., Dominici F., Fortunati E., Kenny J., Puglia D. (2015). Effect of lignin nanoparticles and masterbatch procedures on the final properties of glycidyl methacrylate-g-Poly(lactic acid) films before and after accelerated UV weathering. Ind. Crop. Prod..

[40] Yang W., Weng Y., Puglia D., Qi G., Dong W., Kenny J.M., Ma P. (2020). Poly(lactic acid)/lignin films with enhanced toughness and anti-oxidation performance for active food packaging. Int. J. Biol. Macromol..

[41] Sun Y., Yang L., Lu X., He C. (2015). Biodegradable and renewable poly(lactide)–lignin composites: Synthesis, interface and toughening mechanism. J. Mater. Chem. A.

[42] Thakur V.K., Thakur M.K., Raghavan P., Kessler M.R. (2014). Progress in green polymer composites from lignin for multifunctional applications: A review. ACS Sustain. Chem. Eng..

[43] Boarino A., Schreier A., Leterrier Y., Klok H.-A. (2022). Uniformly Dispersed Poly(lactic acid)-Grafted Lignin Nanoparticles Enhance Antioxidant Activity and UV-Barrier Properties of Poly(lactic acid) Packaging Films. ACS Appl. Polym. Mater..

[44] Chollet B., Lopez-Cuesta J.-M., Laoutid F., Ferry L. (2019). Lignin Nanoparticles as A Promising Way for Enhancing Lignin Flame Retardant Effect in Polylactide. Materials.

[45] Iglesias Montes M.L., Luzi F., Dominici F., Torre L., Cyras V.P., Manfredi L.B., Puglia D. (2019). Design and characterization of PLA bilayer films containing lignin and cellulose nanostructures in combination with umbelliferone as active ingredient. Front. Chem..

[46] Yang W., Fortunati E., Dominici F., Kenny J.M., Puglia D. (2015). Effect of processing conditions and lignin content on thermal, mechanical and degradative behavior of lignin nanoparticles/polylactic (acid) bionanocomposites prepared by melt extrusion and solvent casting. Eur. Polym. J..

[47] Costes L., Laoutid F., Khelifa F., Rose G., Brohez S., Delvosalle C., Dubois P. (2016). Cellulose/phosphorus combinations for sustainable fire retarded polylactide. Eur. Polym. J..

[48] Li H., McDonald A.G. (2014). Fractionation and characterization of industrial lignins. Ind. Crops Prod..

[49] Abdelwahab M.A., Jacob S., Misra M., Mohanty A.K. (2021). Super-tough sustainable biobased composites from polylactide bioplastic and lignin for bio-elastomer application. Polymer.

[50] Toda A., Androsch R., Schick C. (2016). Insights into polymer crystallization and melting from fast scanning chip calorimetry. Polymer.

[51] Androsch R., Iqbal H.M.N., Schick C. (2015). Non-isothermal crystal nucleation of poly (l-lactic acid). Polymer.

[52] Klonos P.A. (2018). Crystallization, glass transition, and molecular dynamics in PDMS of low molecular weights: A calorimetric and dielectric study. Polymer.

[53] Beslikas T., Gigis I., Goulios V., Christoforides J., Papageorgiou G.Z., Bikiaris D.N. (2011). Crystallization Study and Comparative in Vitro–in Vivo Hydrolysis of PLA Reinforcement Ligament. Int. J. Mol. Sci..

[54] Schick C. (2009). Differential scanning calorimetry (DSC) of semicrystalline polymers. Anal. Bioanal. Chem..

[55] Klonos P.A., Bikiaris D.N., Kyritsis A., Schönhals A., Szymoniak P. (2022). Molecular Mobility in Nanocomposites Based on Renewable Semicrystalline Polyesters. Dynamics of Composite Materials.

[56] Cowie J.M.G., McEwen I.J. (1973). Molecular motions in poly(dimethyl siloxane) oligomers and polymers. Polymer.

[57] Sargsyan A., Tonoyan A., Davtyan S., Schick C. (2007). The amount of immobilized polymer in PMMA SiO2 nanocomposites determined from calorimetric data. Eur. Polym. J..

[58] Wurm A., Ismail M., Kretzschmar B., Pospiech D., Schick C. (2010). Retarded Crystallization in Polyamide/Layered Silicates Nanocomposites caused by an Immobilized Interphase. Macromolecules.

[59] Kontou E., Niaounakis M., Georgiopoulos P. (2011). Comparative study of PLA nanocomposites reinforced with clay and silica nanofillers and their mixtures. J. Appl. Polym. Sci..

[60] Purohit P.J., Wang D.-Y., Wurm A., Schick C., Schönhals A. (2014). Comparison of thermal and dielectric spectroscopy for nanocomposites based on polypropylene and Layered Double Hydroxide—Proof of interfaces. Eur. Polym. J..

[61] Tadiello L., D’Arienzo M., Di Credico B., Hanel T., Matejka L., Mauri M., Morazzoni F., Simonutti R., Spirkova M., Scotti R. (2015). The filler–rubber interface in styrene butadiene nanocomposites with anisotropic silica particles: Morphology and dynamic properties. Soft Matter.

[62] Klonos P., Kyritsis A., Pissis P. (2015). Interfacial dynamics of polydimethylsiloxane adsorbed on fumed metal oxide particles of a wide range of specific surface area. Polymer.

[63] Leng J., Kang N., Wang D.-Y., Wurm A., Schick C., Schönhals A. (2017). Crystallization behavior of nanocomposites based on poly(l-lactide) and MgAl layered double hydroxides—Unbiased determination of the rigid amorphous phases due to the crystals and the nanofiller. Polymer.

[64] Klonos P., Dapei G., Sulym I.Y., Zidropoulos S., Sternik D., Deryło-Marczewska A., Borysenko M.V., Gun’Ko V.M., Kyritsis A., Pissis P. (2016). Morphology and molecular dynamics investigation of PDMS adsorbed on titania nanoparticles: Effects of polymer molecular weight. Eur. Polym. J..

[65] Righetti M.C., Gazzano M., Di Lorenzo M.L., Androsch R. (2015). Enthalpy of melting of α′- and α-crystals of poly(l-lactic acid). Eur. Polym. J..

[66] Delpouve N., Saiter A., Dargent E. (2011). Cooperativity length evolution during crystallization of poly(lactic acid). Eur. Polym. J..

[67] Fischer E.W., Sterzel H.J., Wegner G. (1973). Investigation of the structure of solution grown crystals of lactide copolymers by means of chemical reactions. Kolloid-Z. Z. Polym..

[68] Jariyavidyanont K., Du M., Yu Q., Thurn-Albrecht T., Schick C., Androsch R. (2022). Bulk Enthalpy of Melting of Poly(l-lactic acid) (PLLA) Determined by Fast Scanning Chip Calorimetry. Macromol. Rapid Commun..

[69] Aranguren M.I. (1998). Crystallization of polydimethylsiloxane: Effect of silica filler and curing. Polymer.

[70] Pan P., Zhu B., Kai W., Dong T., Inoue Y. (2008). Polymorphic Transition in Disordered Poly(l-lactide) Crystals Induced by Annealing at Elevated Temperatures. Macromolecules.

[71] Tábi T., Hajba S., Kovács J.G. (2016). Effect of crystalline forms (α′ and α) of poly(lactic acid) on its mechanical, thermo-mechanical, heat deflection temperature and creep properties. Eur. Polym. J..

[72] Tarani E., Pušnik Črešnar K., Zemljič L.F., Chrissafis K., Papageorgiou G.Z., Lambropoulou D., Zamboulis A.N., Bikiaris D., Terzopoulou Z. (2021). Cold Crystallization Kinetics and Thermal Degradation of PLA Composites with Metal Oxide Nanofillers. Appl. Sci..

[73] Lu Q., Zhu M., Zu Y., Liu W., Yang L., Zhang Y., Zhao X., Zhang X., Zhang X., Li W. (2012). Comparative antioxidant activity of nanoscale lignin prepared by a supercritical antisolvent (SAS) process with non-nanoscale lignin. Food Chem..

[74] Domínguez-Robles J., Martin N.K., Fong M.L., Stewart S.A., Irwin N.J., Rial-Hermida M.I., Donnelly R.F., Larrañeta E. (2019). Antioxidant PLA composites containing lignin for 3D printing applications: A potential material for healthcare applications. Pharmaceutics.

[75] Wang N., Zhang C., Weng Y. (2021). Enhancing gas barrier performance of polylactic acid/lignin composite films through cooperative effect of compatibilization and nucleation. J. Appl. Polym. Sci..

