# Supplementary material for: Effect of Micro- and Nano-Lignin on the Thermal, Mechanical, and Antioxidant Properties of Biobased PLA–Lignin Composite Films
Source: Polymers (Basel). 2022 Dec 2;14(23):5274. doi: 10.3390/polym14235274 (PMC9737150; doi:10.3390/polym14235274)
Supplement: Supplementary file 1 [file polymers-14-05274-s001.zip › polymers-2072699-supplementary.pdf]

Supporting information for:

Effect of micro- and nano-lignin on thermal, mechanical and antioxidant properties of biobased PLA/lignin composite films

Sofia P. Makri[1,2], Eleftheria Xanthopoulou[2], Panagiotis A. Klonos[2,3], Alexios Grigoropoulos[1], Apostolos Kyritsis[3], Konstantinos Tsachouridis[4], Antonios Anastasiou[4], Ioanna Deligkiozi[1], Nikolaos Nikolaidis[2] and Dimitrios N. Bikiaris [2,*]

[1] Creative Nano PC, 43 Tatoiou, Metamorfosi 14451, Athens, Greece
[2] Department of Chemistry, Laboratory of Polymer Chemistry and Technology, Aristotle University of Thessaloniki, GR-541 24, Thessaloniki, Greece
[3] Department of Physics, National Technical University of Athens (NTUA), Zografou Campus, 15780, Athens, Greece
[4] Department of Chemical Engineering and Analytical Science, University of Manchester, Manchester M1 3AL, UK

* Correspondence: dbic@chem.auth.gr (D.N.B.)

Characterization of Lignin/ Nanolignin

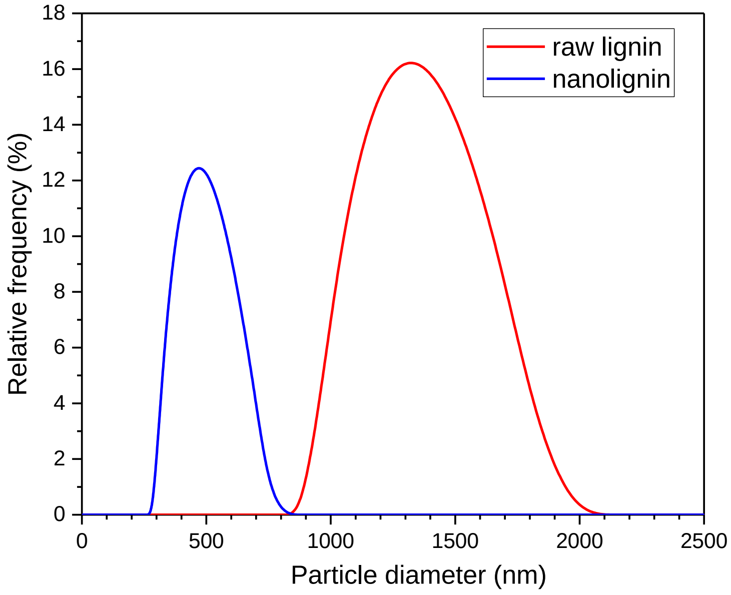

Figure S1. Particle size distribution curve of raw soda lignin and nanolignin corresponding to hydrodynamic diameter of 2.38 μm (polydispersity index, PDI = 0.29) and 524 nm (PDI = 0.161), respectively.

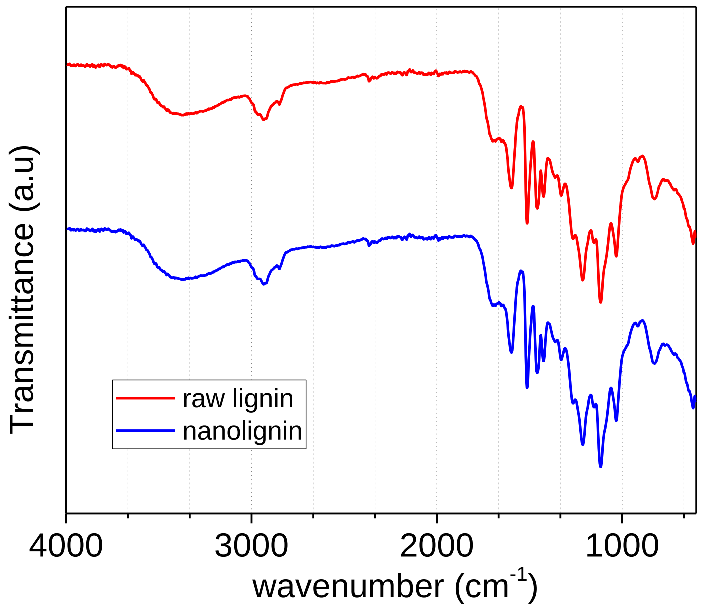

Figure S2. FTIR spectra of soda lignin and nanolignin.

Scanning Electron Microscopy (SEM)

<table>
<tr><th colspan="2">PLA neat</th></tr>
<tr><td colspan="2">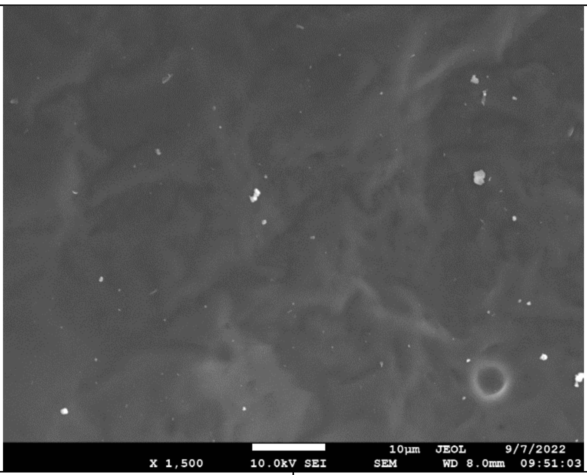
</td></tr>
<tr><td>PLA-0.5%wt L</td><td>PLA-0.5%wt NL</td></tr>
<tr><td>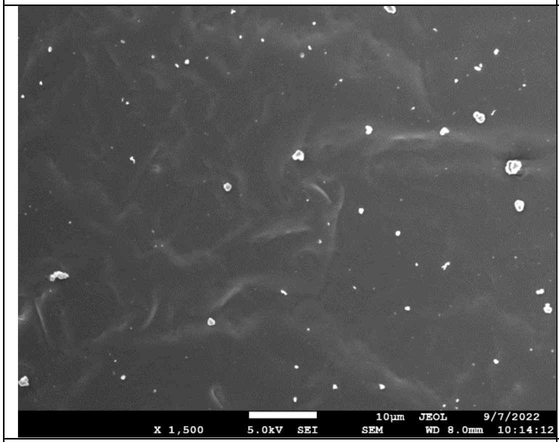
</td><td>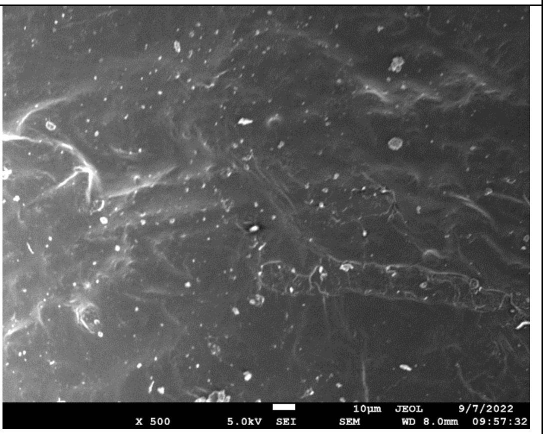
</td></tr>
<tr><td>PLA-1.0%wt L</td><td>PLA-1.0%wt NL</td></tr>
<tr><td>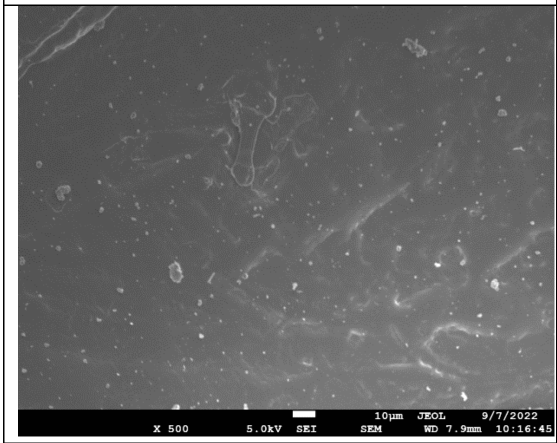
</td><td>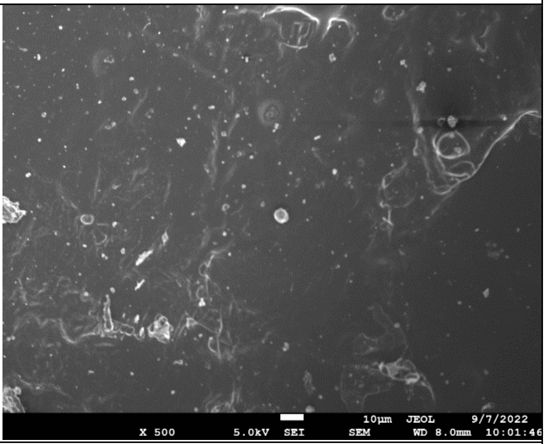
</td></tr>
<tr><td>PLA-2.5%wt L</td><td>PLA-2.5%wt NL</td></tr>
</table>

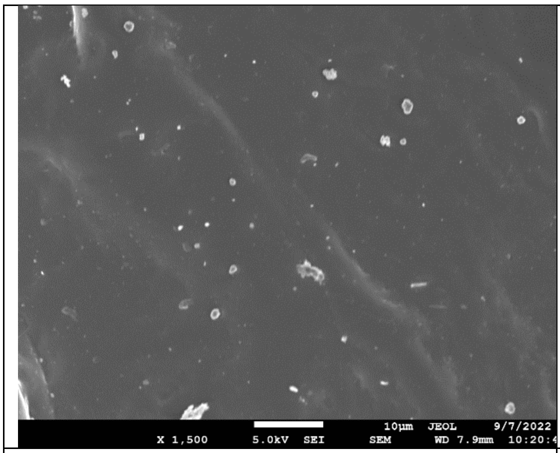

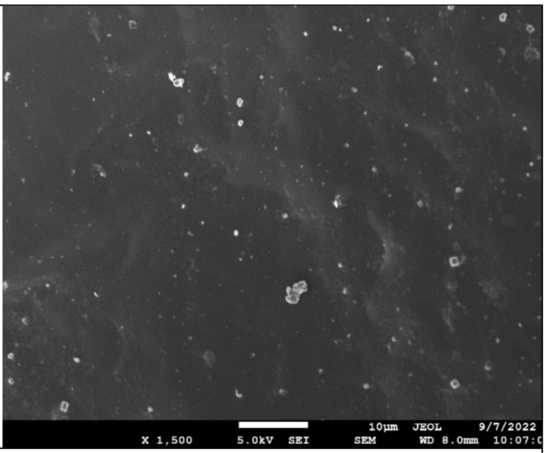

PLA-5.0%wt L | **PLA-5.0%wt NL**

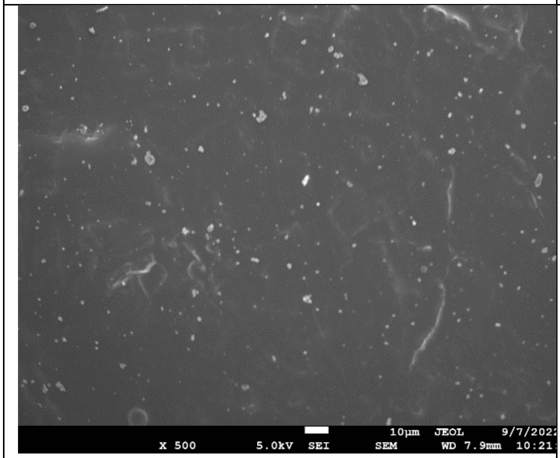

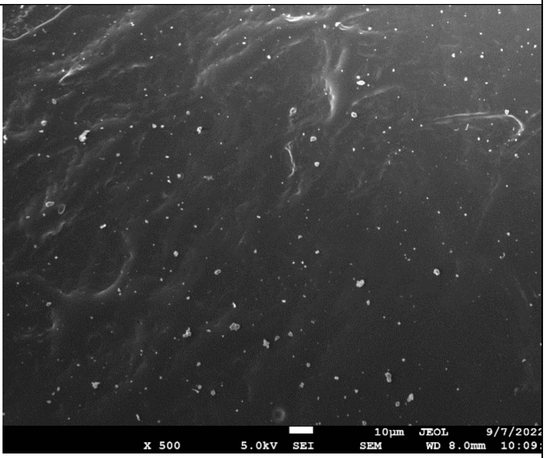

Figure S3. SEM images of PLA-L/ NL composites containing different L/NL content.

Mechanical properties

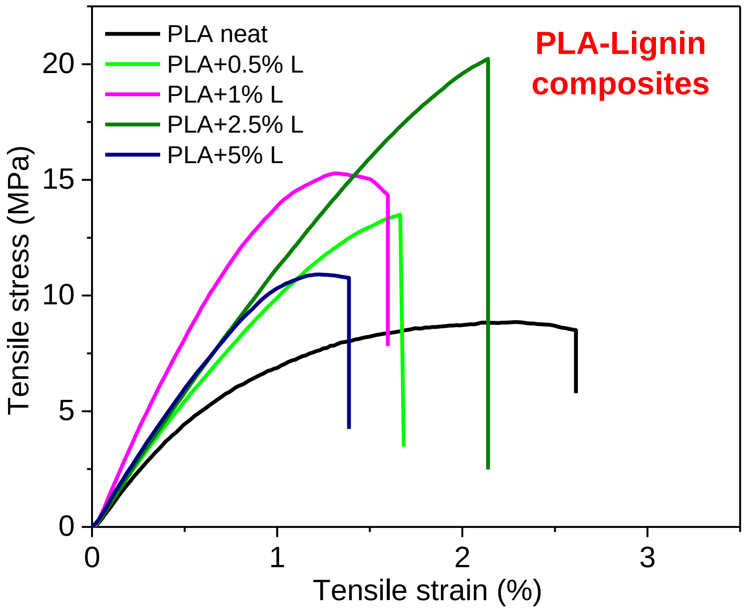

Figure S4. Tensile stress-strain curves of PLA-L composites.

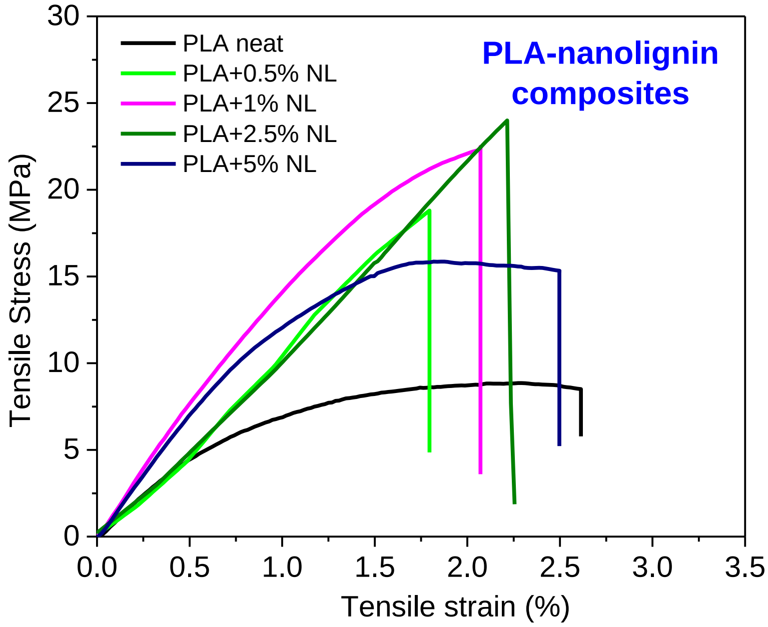

Figure S5. Tensile stress-strain curves of PLA-NL composites.

Antioxidant activity of composites

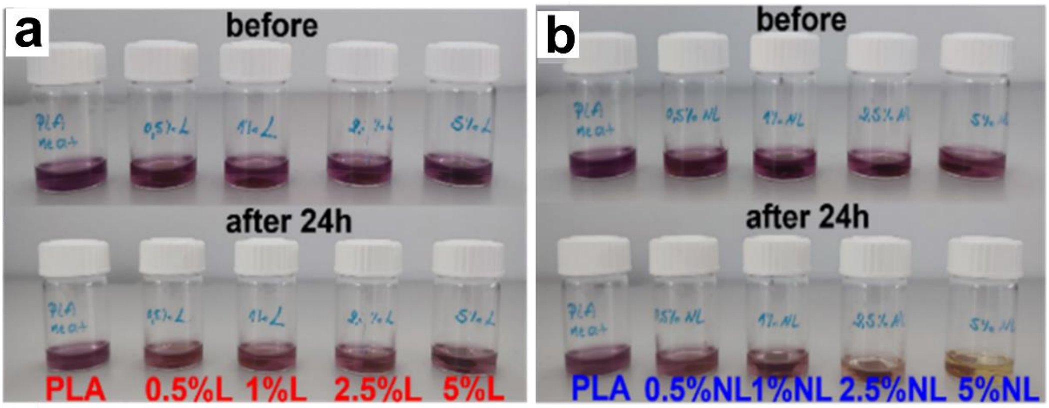

Figure S6. Qualitative antioxidant capacity of composites- Photographs of the DPPH / ethanol solution for different PLA-Lignin (a) and PLA-Nanolignin (b) composites after 24 h exposure to PLA-L/NL films.
